# Supplementary material for: Intra-Laboratory Evaluation of DNA Extraction Methods and Assessment of a Droplet Digital PCR for the Detection of Xanthomonas citri pv. citri on Different Citrus Species
Source: Int J Mol Sci. 2022 Apr 29;23(9):4975. doi: 10.3390/ijms23094975 (PMC9105834; doi:10.3390/ijms23094975)
Supplement: Supplementary file 1 [file ijms-23-04975-s001.zip › ijms-1642514-supplementary-done.pdf]

International Journal of
Molecular Sciences

Table S1. Real-time PCR results of 20 healthy randomly selected samples for each plant matrices/DEM combination. Ct values and/or inconsistencies between the two technical replicates (i.e. high Ct value/NA) are reported.

	Ct value							
	**Plant**		**Mericon**		**Ctab**		**Quick**	
**Lemon fruit**	37.43	NA	39.17	NA	40.11	NA	39.46	39.59
	39.65	NA	38.11	NA	37.66	NA		
	39	NA	36.47	NA	38.4	NA		
	39.4	NA	37.31	NA	38.5	NA		
			35.63	NA	39	38.8		
			35.04	NA	37.8	37.9		
					36.2	36.6		
					37.4	40		
					37.3	38.7		
**Orange fruit**	37.82	NA	37.02	NA	37.98	NA	40.08	NA
	36.93	NA	37	NA	36.47	NA	42.77	NA
			37.43	NA	41.7	NA	41	NA
			38.18	NA	37.26	NA	39	NA
					40.53	NA	41.56	NA
					37.22	NA		
					38.42	38.36		
					42.44	NA		
**Lemon Leaf**	38.99	NA	37.21	NA	38.25	NA	38.31	NA
	37.51	35.93	37.66	37.14	38.55	NA		
	37.92	NA	37.06	NA	38.62	NA		
	37.68	NA	42	NA	36.11	36.02		
	38.19	NA	38.32	NA	37.07	38.11		
	38.78	NA	37.24	36.81	36.4	NA		
	39.28	NA	37.02	NA	38.61	NA		
	37.8	NA			38.31	NA		
	36.42	NA						
